# Game Theory Paradigm: A New Tool for Investigating Social Dysfunction in Major Depressive Disorders

**DOI:** 10.3389/fpsyt.2015.00128

**Published:** 2015-09-15

**Authors:** Yun Wang, Liu-Qing Yang, Shu Li, Yuan Zhou

**Affiliations:** ^1^Key Laboratory of Behavioral Science and Magnetic Resonance Imaging Research Center, Institute of Psychology, Chinese Academy of Sciences, Beijing, China; ^2^University of Chinese Academy of Sciences, Beijing, China

**Keywords:** major depressive disorder, social dysfunction, game theory, ultimatum game, social interaction

## Abstract

Social dysfunction is a prominent source of distress and disability in patients with major depressive disorder (MDD) but is commonly omitted from current clinical studies, although some researchers propose an evolutionary strategy to understand these negative outcomes. Limited knowledge about the neural basis of social dysfunction in MDD results from traditional paradigms, which lack insights into social interactions. Game theoretical modeling offers a new tool for investigating social-interaction impairments in neuropsychiatric disorders. This review first introduces three widely used games from game theory and the major behavioral and neuroimaging findings obtained using these games in healthy populations. We also address the factors that modulate behaviors in games and their neural bases. We then summarize the current findings obtained by using these games in depressed patients and discuss the clinical implications of these abnormal game behaviors. Finally, we briefly discuss future prospects that may further elucidate the clinical use of a game theory paradigm in MDD.

## Introduction

Major depressive disorder (MDD) is a common psychiatric disorder that is characterized by a ­persistent and overwhelming feeling of sadness and a consistent loss of interest or pleasure in normal activities (1). Frequently, MDD is associated with significant and pervasive impairments in social functioning, defined as an individual’s ability to perform and fulfill normal social roles (2). Assuming that the associated negative outcomes are the result of disease rather than a functional adaption, understanding the mechanisms of social dysfunction is essential for preventing or ameliorating the associated negative outcomes. Researchers have used social cognition as a lens for investigating whether MDD patients can adaptively interpret the social information that is a crucial part of successful social interaction. Using traditional paradigms, such as theory of mind (ToM) tasks and facial emotional processing tasks, researchers have found impairments in MDD patients’ ability to understand and respond to other people’s thoughts, feelings, reactions, concerns, and motives (3–5). However, these studies often investigated the social cognition of MDD patients using tasks that are in non-social-interaction contexts and may not reflect the dynamic and unique nature of real-life interactions with the environment, in particular the aspects of their own personal environment that may be troubling them. Therefore, traditional social cognition paradigms make it difficult to study whether MDD patients can appropriately respond to social events in dynamic and context-appropriate ways (6).

A game theory paradigm can provide a new tool for investigating social-interaction impairments in neuropsychiatric disorders (7). Game theory (8) is a collection of mathematical models that attempt to study decision behaviors where several players must make choices that potentially affect the interests of other players. It provides a prolific source of both interactive tasks and well-specified models for investigating social exchange. It also allows for complex and ecologically more valid contexts, within which social functioning can be examined, to be created (9). An interactive game theory paradigm may help to identify suboptimal choices and maladaptations associated with MDD and thus may potentially provide a powerful tool for discovering candidate biomarkers or endophenotypes in MDD (6).

In this article, we first briefly introduce three widely used games from game theory and present the major behavioral and neuroimaging findings in healthy populations. Factors modulating behaviors in these games, especially in the ultimatum game (UG), and their neural bases are also addressed. Then, we summarize the major research advances obtained by using a game theory paradigm in studies of depressed patients and discuss the clinical implications of these differences between the game behaviors of people with MDD and those of control groups. Finally, we briefly discuss current challenges and potential research directions that may help to further elucidate the clinical use of the game theory paradigm in MDD.

## Research Advances in Game Theory Studies in Healthy Populations

A set of abilities essential for behaving in accord with other people and for interacting with them compose our social functioning. Of these behaviors, strategic bargaining and reciprocal exchange are two social-interaction behaviors that have consequences. The field of economic game theory provides a set of useful, widely used tasks that allow the investigation of such behaviors in a social-interaction context (10). The UG has often been used to study strategic bargaining behavior, and the trust game (TG) and the prisoner’s dilemma game (PDG) have been applied to the study of reciprocal exchange. By establishing bargaining and reciprocal exchange games that are played with partners, game theory provides a useful foundation for the study of social functioning in real, consequential social scenarios.

### The ultimatum game

**Figure 1:**
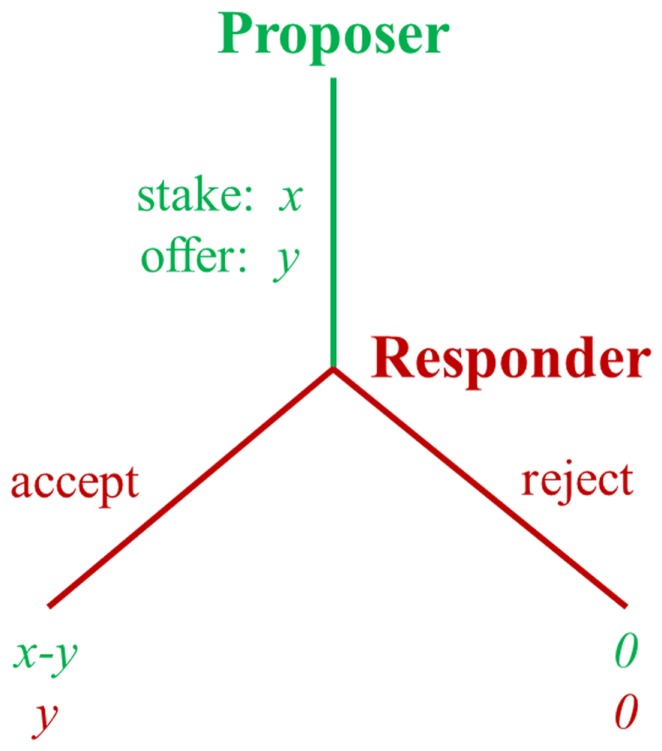
**Schematic diagram of the ultimatum game**.

The UG (11) is often used to examine responses to fairness. In the UG, a proposer first makes a proposal about how to divide a fixed amount of money (i.e., the stake). Then the responder has to decide whether to accept or reject the proposal without negotiation. If the responder accepts, the proposed split is realized. If he/she rejects, neither of them receives anything (Figure 1).

In the UG, the payoff-maximizing strategy for the responder is to accept all non-zero offers, and for the proposer to make the smallest possible offer. However, contrary to this prediction, experiments from different countries consistently reveal that people do not always pursue their own maximum short-term profits (10). Empirically, proposers typically offer about 40% of the money and responders usually reject unfair offers. About 50% of all unfair offers (defined as 20% or less of the stake) are typically rejected and rejection rates increase as offers become less fair (12).

To understand the mechanisms underlying the responders’ rejection of unfair offers in the UG, many researchers have investigated the neural basis of such behavior by using functional magnetic resonance imaging (fMRI). The seminal work of Sanfey et al. (13) found that unfair offers elicited activity in brain areas related to emotion (anterior insula), cognition [dorsolateral prefrontal cortex (PFC)], and cognitive conflict [anterior cingulate cortex (ACC)]. By finding a correlation between the anterior insula and acceptance rates, this study strengthened our understanding of the role of emotional processes in human decision-making, as indicated by the UG. Since then, a number of neuroimaging studies have verified or extended the findings of Sanfey et al. (13) by identifying potential neural substrates of unfairness and decisions to reject vs. accept an offer [e.g., (14, 15)]. Gabay et al. (16) and Feng et al. (17) both used meta-analyses to summarize published fMRI articles based on the UG. Each of these meta-analyses revealed consistent activations in the anterior insula, ACC, and supplementary motor area (SMA) in response to unfair offers. Gabay et al. (16) also showed that robust activations in the ACC, SMA, right middle frontal gyrus (MFG), and lentiform nucleus occurred when the participants decided to reject rather than accept UG offers. In addition, fair offers in the UG led to consistently stronger activations in the bilateral ventromedial PFC, posterior insula, the left posterior cingulate cortex (PCC) and precuneus, and the right inferior temporal gyrus compared with the activations found in those areas in unfair offers (17). Accordingly, Feng et al. (17) suggested that two systems, a reflexive and intuitive system (System 1) and a reflective and deliberate system (System 2), are involved in the fairness-related decision-making. System 1 includes the anterior insula, amygdala, and ventromedial PFC, whereas System 2 includes the dorsal ACC, ventrolateral PFC, dorsomedial PFC, and dorsolateral PFC. The intuitive system is involved in rapidly evaluating norm violations and the deliberate system is involved in integrating both self-interest and social norms to regulate the intuitive system to permit more flexible decision-making (17).

Behavioral studies have also shown that proposers in the UG usually propose fair distributions, but the psychological mechanism and neural basis behind this fair behavior remain unclear. One solution is to compare the proposers’ fair behaviors and their brain activations in the UG with those in a dictator game (DG). The differences may provide some insights into proposers’ decisions in the UG. In the DG, the proposer makes a similar type of split, but the responder has no choice but to accept the offer. As in the UG, the standard economic solution to the DG is that the rational and self-interested proposer would always offer the minimal amount of money to the responder. However, empirical evidence demonstrates that proposers share certain portions of their money (18). In the DG, the responder has no option but to accept the offer, so any fair behavior by the proposers in the DG may reflect different motivations than those in the UG. Weiland et al. (19) examined the brain activity that contributes to fair and unfair behaviors of proposers in the UG and DG with the goal of exploring whether egoistic and altruistic motives of the proposers affect fairness differently in the two games. They found that fair offers in the UG were related to enhanced activity in the medial prefrontal cortex (MPFC), dorsolateral PFC, medial orbitofrontal cortex, posterior parietal cortex, and striatum, brain areas which are involved with reward and with the ToM. This corroborates the idea that egoistic motives, conjecturing the behaviors of others, and making acceptable offers to get self-beneficial results, including long-term results made possible by pan-cultural systems of direct and indirect reciprocity, are primarily responsible for fair offers in the UG. However, fair offers in the DG were related to increased activity in the dorsal ACC and the PCC, which are related to cognitive conflict. This supports the idea that in the conflict between altruism and self-profit, altruistic motives primarily drive the proposer to make fair offers in the DG (19). Zheng and Zhu (20) also investigated the differences in neural activity between a proposer’s decision-making in the UG and the DG and further validated the findings of Weiland et al. (19). In addition, some researchers explored this problem from the perspective of norm compliance. Because of differences in the rules between these two games, researchers have considered that a proposer’s fair behavior in the DG results from voluntary norm compliance, whereas fair behavior in the UG has been deemed sanction-induced norm compliance. Ruff et al. (21) investigated the biological mechanisms underlying fair norm compliance in the DG and UG using transcranial direct current stimulation (tDCS), in which neural excitability in the right lateral PFC was enhanced with anodal tDCS, reduced with cathodal tDCS, or left unaltered by a sham/placebo tDCS as a control. The results showed that the right lateral PFC is involved in both voluntary and sanction-induced norm compliance, but they affected it in opposite ways. Specifically, the amount of money transferred in the DG decreased during anodal tDCS and increased during cathodal tDCS, relative to the sham condition; however, the amount of money transferred in the UG increased during anodal tDCS and decreased during cathodal tDCS, relative to the sham condition. These results suggest that these two forms of norm compliance involve distinct neural circuits; in particular, the right lateral PFC seems to play a fundamentally different role in voluntary and sanction-based norm compliance. In brief, by comparing with the DG, researchers examined the motivations and neural bases of fair behavior in the UG, and this endeavor identified the key role of strategic motivations and of the PFC when proposers make fair offers.

### The trust game

**Figure 2:**
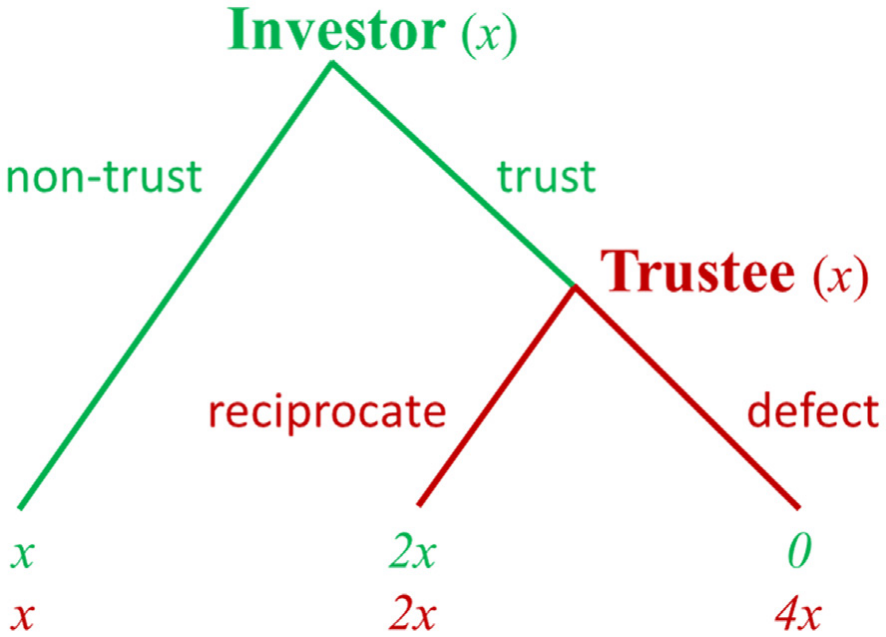
**Schematic diagram of the trust game**. Note: at the beginning, each player is endowed with equal amounts of money [e.g., (x, x)]. The investor can choose “non-trust” and quit the game with a small payoff for both players [i.e., (x, x)] or can choose “trust” to continue the game. If the investor chooses “trust” and invests his money, this money that the investor invests to the trustee is multiplied by some factor (e.g., 3). The trustee then can choose “reciprocate” and return some money back to the investor, giving them both a higher payoff [e.g., (2x, 2x)] or choose “defect” and keep the additional 3x for himself, resulting in an even larger payoff to the trustee and a payoff of 0 to the investor [e.g., (0, 4x)]. By substituting different payoff numbers, different incentives for cooperation can be studied.

The TG has been used to study reciprocal exchanges in economic transactions. In the TG (22), initially an investor must decide how much money to invest with the trustee. If invested, the money is multiplied by some factor, and then the trustee has the option to return any proportion of the multiplied amount to the investor. If the trustee honors trust and sends money back, both players can end up with a higher monetary payoff than was originally obtained. However, if the trustee abuses trust and possesses all the money, the investor takes a loss (Figure 2). Thus, from the perspective of trustee, the classical TG is in some ways similar to the DG in that the trustee becomes a dictator once the investor has surrendered their money.

In the classical TG, because the investor and trustee interact only once, the game theory prediction is that a rational trustee will not honor the trust from the investor. Accordingly, the investor, realizing this, will invest nothing in the transaction. In spite of these theoretical predictions, empirical studies found that most of the investors do send some money back to the trustees, and the trust is reciprocated in general (22). Previous investigations of the TG indicate that different neural systems, such as reward, ToM, and social attachment systems, are critical for understanding the neurobiological basis of trust and reciprocity (23–27). In one fMRI study, the participants played an economic TG with both human and computer counterparts for cash rewards (25). Within the group of subjects who received the highest cooperation scores, the prefrontal regions were more active when the subjects were playing with a human than when they were playing with a computer following a fixed and known probabilistic strategy. In another study, the fMRI results showed that the anterior MPFC was more active when participants defected than when they reciprocated (27). Krueger et al. (24) explored the neural mechanism of trust in a non-anonymous, repeated TG. The results showed that two brain systems may contribute to building the first player’s trust. A personal “unconditional” trust system involving early activation of the anterior region of the rostral MPFC (mentalizing) is followed by a later activation of the septal area, a region that has been indicated to modulate various social behaviors. A second “conditional” trust system does not use the mentalizing system but use the reinforcement learning system (ventral tegmental area) to build trust. Krueger et al. (23) did further analyses of the experiment reported in Krueger et al. (24) and explored the shared and specific networks for trust and reciprocity. They found that trusting and reciprocating behaviors draw on common neural systems of mentalizing (anterior rostral MPFC and temporoparietal junction) and empathy (anterior insula). In addition, an evaluation system for prospective outcomes (frontopolar cortex) was specifically involved in trusting behavior. Together, these studies provide insight into how several brain regions work together when individuals choose to trust others and, further, to reciprocate trust. In this way, they have extended our knowledge of the neural bases of trust and reciprocity in reciprocal exchanges.

### The prisoner’s dilemma game

**Figure 3:**
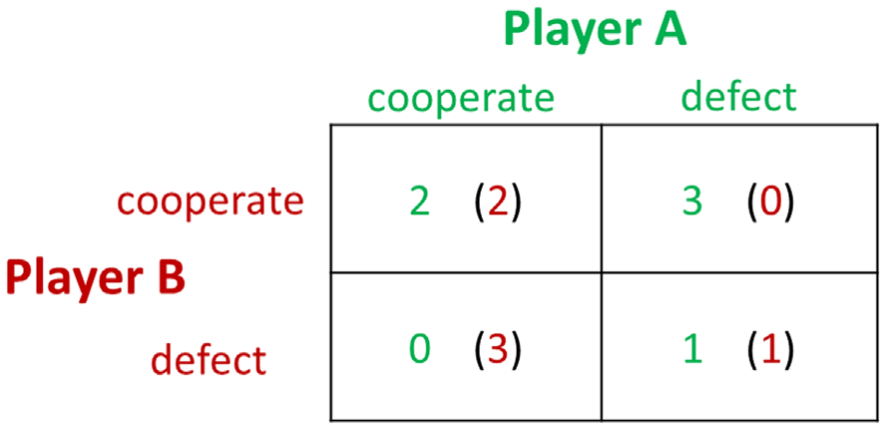
**An example of a payoff matrix in the prisoner’s dilemma game**.

The PDG was created to study conditions necessary to the evolution of cooperation in the context of players having conflicts of interest, in which there would, therefore, be a temptation to defect or cheat rather than to cooperate. The PDG (28) confronts each of two players with the same options: cooperate or defect. There are four possible outcomes of a round: player A and player B cooperate (CC), player A cooperates and player B defects (CD), player A defects and player B cooperates (DC), or player A and player B defect (DD). The payoffs for the outcomes are arranged such that DC > CC > DD > CD, and CC > (CD + DC)/2. Figure 3 is an example of a payoff matrix in the experimental design of PDG studies. Each block of the payoff matrix represents a different outcome of a social interaction.

In the PDG, the interaction between the two players determines their payoffs. The largest payoff to a player occurs when he or she defects and the partner cooperates, and the worst outcome occurs when the player cooperates while the partner defects. Mutual cooperation brings a modest payoff to both players, while mutual defection yields a lesser amount to each. The game theory prediction for the non-iterated PDG among strangers is mutual defection. However, empirical studies confirm that players exhibit more trust than expected, with mutual cooperation occurring approximately half of the time (28). Researchers from the fields of social neuroscience and neuroeconomics have begun to study the neural underpinnings of the PDG. In an fMRI study (29), reward-related regions, such as the nucleus accumbens, caudate nucleus, and ventromedial frontal/orbitofrontal cortex, were detected when the participants engaged in mutual cooperation. In addition, activation in the rostral ACC increased when subjects chose to cooperate after their partner had cooperated in the previous round, corresponding to the role of the ACC in detecting cognitive conflict. In a follow-up fMRI study implementing repeated one-shot PDGs, Rilling et al. (30) replicated their earlier findings and strengthened the conclusion that subjects in repeated PDGs learn to cooperate by using neural-based reinforcement learning strategies. Subsequently, Rilling et al. (31) further investigated the neural responses to non-reciprocation of cooperation in the PDG. The results showed that unreciprocated cooperation was associated with greater activity in the bilateral anterior insula, left hippocampus, and left lingual gyrus, compared with reciprocated cooperation. Functional connectivity between the anterior insula and the lateral orbitofrontal cortex in response to unreciprocated cooperation predicted subsequent defection. This study indicates that the anterior insula and the lateral orbitofrontal cortex may be the cause of negative feeling states that bias subsequent decisions against cooperation with a non-reciprocating partner.

## Factors Modulating Game Behaviors and Their Neural Bases

The wealth of data produced by the game theory paradigm is of great interest to researchers from different fields. However, because many contextual factors and experimental parameters can affect game behavior, comparisons between studies are complicated. In order to have a clear understanding of the cognitive mechanisms of social interactions in the game theory paradigm and their neural bases, we must remain aware of the multiple sources of potential confounds. In this section, we will focus on the neural substrates of the factors that modulate game behaviors. The factors modulating behaviors in the UG and their neural bases have been investigated in numerous studies. However, few neuroimaging studies have addressed the underlying neural substrate of the factors modulating behaviors in the TG and PDG. Thus, this section will primarily summarize the neuroimaging studies that investigated factors affecting UG behavior and their neural bases one by one but only briefly summarize the behavioral studies that explored the factors modulating behaviors in the TG and PDG.

### Factors modulating UG behaviors and their neural bases

In the UG, increasing evidence has shown that individuals’ social decisions are not only affected by the fairness of the offer itself, but also modulated by various social factors. Researchers have investigated the factors affecting UG behaviors, such as stake size, gain–loss contexts, gain–loss frames, group opinion, social status, and emotion, along with their neural bases.

#### Stake Size

A unique feature of human beings is a strategically contingent compliance with social norms even though this normative decision means curbing short-term self-interest. However, sometimes money talks. Previous behavioral studies that used the UG showed deviations from the fairness-related normative decision (rejection of unfair proposals) as a result of high monetary incentives. For high stakes, responders tend to reduce the threshold below which they reject proposals (32–37). In order to investigate the neural substrates underlying this deviation, our group conducted an fMRI study using a revised UG in which fairness and a proposed monetary amount varied orthogonally (38). Consistent with previous reports, we found that the rejection rates for unfair proposals with a high stake size were significantly lower than those for unfair proposals with a low stake size. This behavioral deviation from the normative decision was reflected in the participants’ brain activations. The fMRI results showed that the fairness-related activations of the bilateral insular cortices and the right lateral PFC were modulated by monetary incentives. Additionally, inter-individual differences in the modulation effects in the left inferior frontal gyrus (IFG) accounted for inter-individual differences in the behavioral modulation effect, as measured by the rejection rate. This study provided neural evidence for the modulation of fairness by the size of a monetary incentive and also for inter-individual differences in the deviation from fairness-related normative choices.

#### Gain–Loss Contexts

Social behaviors in studies using the UG have been widely investigated in the gain context, i.e., when players split a certain amount of money as their gains (11, 13, 39). However, the problem of negotiating over losses, such as the liquidation of a bankrupt company, is as unavoidable and problematic as the problem of negotiating over gains. Recent studies have tried to probe the potential effect of gain and loss contexts on players’ decisions in the UG. In the loss context, the proposer and responder need to pay a sum of money. Acceptance by the responder will lead to the suggested division of the payment and rejection will result in a complete loss for both players. Behavioral studies have revealed that responders assigned lower fairness ratings and rejected more offers in the loss context compared with the gain context (40, 41). Researchers also examined the neural mechanisms underlying rejections of unfair losses and unfair gains in the UG. Guo et al. (42) found that the left dorsolateral PFC, bilateral anterior insula, ACC/anterior middle cingulate cortex, and bilateral dorsal striatum were activated when comparing rejection with acceptance in the loss but not the gain context. Wu et al. (43) found that the positive correlation between fairness and activation in the ventral striatum was reduced, while the negative correlations between fairness and activation in the dorsolateral PFC were enhanced in the loss context. Additionally, rejection-related dorsal striatum activation was higher in the loss context. Together, the results indicated that participants may undergo more unfairness and a stronger desire to punish social norm violations, inducing more fairness-related neural activities in the loss context.

#### Gain–Loss Frames

The way the information is formulated, such as gain or loss, has been found to affect people’s risky decisions (44, 45). The way an offer is framed may well affect decision-making in the UG. Leliveld et al. (46) manipulated giving and taking situations for UG proposers by putting chips on the proposer’s or the responder’s side of the table. They found that the chips allocated to the responders were highest in the taking UG and lowest in the giving UG. Recent studies also investigated the framing effect on the responder’s decision behavior by manipulating the instructions or the experimental design. In a psychophysiological study, researchers found a defense response (increased heart rate and skin conductance) and a higher rejection rate under the loss frame than under the gain frame (47). The behavioral finding of Wu et al. (48) repeated the above result, and the event-related potentials (ERPs) (P300) were more positive in the “gain” than in the “loss” condition. Although Tomasino et al. (49) did not find any significant effects related to gain–loss frames, neuroimaging data revealed an interaction of frame by response, which showed increased activation in the right rolandic operculum/insular cortex and the anterior cingulate, among other regions, when accepting vs. rejecting in the “loss” frame, as compared to accepting vs. rejecting in the “gain” frame. Additionally, the left occipito-temporal junction was activated for “loss” vs. “gain” for fair offers, consistent with the observation that the same offer could be made unpleasant by the presence of a “loss” frame. These studies extended the current understanding of the neural substrates of UG behavior by exploring the formulations of information processing that are sensitive to gain–loss frames.

#### Group Opinion

When an individual’s actions conflict with those of the group to which they belong, they may alter their initial behavior to comply with the group norm. This phenomenon is known as “social conformity” (50, 51). Individuals’ choices in a monetary game could also be modulated by their peers’ opinions. Grosskopf (52) used a modified UG, in which one proposer faced three responders. The results showed that the rejection rates in the UG with competitive responders were significantly lower than those in the traditional case. Wei et al. (53) further investigated the response of responders to group opinion in the UG while measuring their brain activity. In this study, after the subject made his/her initial choice (accept or reject an offer) in each round, the choices from four other peers, which could be incongruent, moderately incongruent, or congruent with his/her choice, were presented to him/her. Next, the participant was given a second chance to decide how to respond to the same offer. They found that the participants changed their choices when these choices conflicted with the collective opinion of the group, especially in unfair treatment situations. The neuroimaging data revealed that incongruence with group norms activated brain regions, such as the insula, middle temporal gyrus, MFG, inferior parietal lobule, MPFC, and precuneus, that are involved in norm violations and behavioral adjustment. This study indicated that a strong group consciousness motivated individuals to adopt the opinions of the other group members, and then change their initial choices and conform to the group norm. These findings contributed to recent research exploring the neural mechanisms of violations of social norms and provided information about the neural basis of conformity behavior in an economic game.

#### Social Status

Social status, or social rank, refers to an individual’s relative position in terms of wealth, ability, education, stature, or profession in a hierarchy (54). As a highly pervasive principle of social organization found in many species (55), social hierarchy affects the way we see others and ourselves. Individuals with a high standing often have preferential access to the resources vital to reproduction and survival, including food, land, information, power, and potential mating partners; they also have more power or influence over individuals of lower standing (56). Therefore, social status may influence the way we engage in wealth allocation. Using ERPs, Hu et al. (57) investigated the ways that social status modulated acceptance rates and neural responses to offers in the UG. In this study, they used an interactive rank-inducing task to dynamically manipulate the participants’ social status over time, and then asked the participants to act as responders in the UG while their ERPs were simultaneously recorded. The behavioral results showed that the participants were less likely to accept unfair offers when they were endowed with a high status than with a low status. When the participants actually received unfair offers, the late positivity potential (400–700 ms) was more positive in the high status condition than in the low status condition, suggesting an increased arousal for unfair offers in high status individuals. These findings suggest a strong role of social status in modulating individual behavioral and neural responses to fairness.

#### Emotion

An individual’s emotion plays an important role in social decision-making (58). To investigate the effects of emotion on fairness behaviors in the UG, researchers have tried to manipulate individuals’ moods to study the impact of incidental moods on decisions. Harlé and Sanfey (59) induced two basic emotional states (amusement or sadness) which were compared with a neutral-emotion control. They found that higher sadness resulted in lower acceptance rates of unfair offers, whereas induced amusement was not associated with any significant bias in decision-making. Harlé (60) further investigated how such biases are implemented in the brain. Neuroimaging data revealed that unfair offers elicited brain activations related to aversive emotional states and somatosensory integration (anterior insula) and to cognitive conflict (ACC) when in a sad emotional state. Sad participants also exhibited a diminished activation in the ventral striatum, a region associated with reward processing. In addition, insular activation mediated the relationship between sadness and decision bias, demonstrating that mood states can be integrated at the neural level to bias decision-making. Other researchers also found that emotion regulation (61, 62) and different emotion regulation strategies (63) can change the behavioral pattern of responders and their brain activations in the UG. Notably, these studies have provided significant information for our understanding of the effects of emotions on socioeconomic decisions.

In summary, researchers have investigated the factors modulating UG behaviors and their neural bases from a variety of perspectives. These endeavors have deepened our understanding of game behavior in the UG and indicated that people’s fair behavior may be affected by many social factors. Importantly, however, the number of previous studies that have explored the impact of certain factors (such as stake size or social status) is relatively small and the behavioral results of some factors (such as the framing effect) have been inconsistent. Thus, further research is still needed to probe the effects of these factors on UG behavior.

### Factors modulating TG behaviors

Researchers who use the TG have also continuously focused on the parameters that could potentially affect the results and therefore should be taken into account when designing such tasks. Recently, Tzieropoulos (26) reviewed the previous literature and clustered the factors that modulated trust when subjects played the role of the investor into six main factors: (1) The trustee. Specifically, cues about the trustee (e.g., emotion, ethnicity, and facial attractiveness) can have a great impact on the investment decision (64, 65). (2) The administration of the game (single-shot or repeated interactions). Multi-rounds allow the investor to test more parameters that can influence trust than do single interactions. For example, the investor can form an opinion about the trustee’s reputation based on the trustee’s responses across repeated interactions (66). Previous studies have shown that multi-round versions of the TG involve learning and adaptive processes that are rarely obtained during single interactions, although the learning effects can be diminished by other available information (24). (3) The impacts of hormones and genetics. Several behavioral studies have found that natural and manipulated oxytocin (OT) levels have a great influence on trust in the TG (67–69), and genotyping studies have indicated links between the OT receptor gene OXTR and trust behavior in the TG (70). For a detailed review on the effect of OT on TG behavior, see Bartz et al. (71). (4) Inter-individual differences. These can also bring about significant behavioral differences in the TG. For example, recent experience of a traumatic event is considered the strongest factor associated with low trust, followed by belonging to minorities (including being a woman), being economically unsuccessful in terms of income and education, and living in a racially mixed community with a high degree of income disparity (72). (5) Time. It has been reported that spontaneous and rapid decisions can lead to more investments in the TG (73). Thus, time limitation should be carefully taken into consideration when designing cooperative games. (6) Other experimental settings. Other variations in the experimental setup, such as the freedom of choices of the investor and the trustee, the multiplication of the amount, and the use of real money or not, may also affect behaviors. In addition, recently some researchers found that the gain–loss domain (74), reciprocity expectation (75), and feedback (76) impact investor behavior. All these factors have to be carefully considered when using cooperative games.

Compared with investors, only a very few studies have specifically focused on trustees. Knoch et al. (77) manipulated the trustee’s reputation by providing the investor a summary of the trustee’s previous decisions and found that trustees in the reputation condition returned on average 43.8% of the money, but just 24.9% in the anonymous condition. So far, only a few neuroimaging studies have addressed the underlying neural substrate of the factors that modulate trust. In summary, the factors mentioned above remind researchers of how subtle changes can affect behaviors when studying social decision-making.

### Factors modulating PDG behaviors

The factors modulating cooperativeness in the PDG have also been explored and can be clustered into five major categories: (1) Individual features. Shinada and Yamagishi (78) found a negative relationship between physical attractiveness and cooperative behavior among young men but not among older men or women. Reed et al. (79) found that pleasant facial expressions were predictive of cooperative decisions within dyads; whereas contemptuous facial expressions were predictive of non-cooperative decisions within dyads. These studies indicated that ecological factors and individual characteristics can have a large impact on an individual’s decision-making in the PDG. (2) Experimental termination rules. Normann and Wallace (80) compared three different experimental termination rules in four treatments: a known finite end, an unknown end, and two variants with a random termination rule. They found that the termination rules did not significantly affect average cooperation rates. However, the termination rules may influence cooperation over time and end-game behavior. Specifically, an (expected) longer length of the game significantly increased cooperation rates. (3) Serotonin. Wood et al. (81) found that serotonin plays a crucial role in cooperative behavior. (4) OT and social value orientation. Declerck et al. (82) found a joint effect in individuals with a proself social value orientation between OT and his/her personality trait social value orientation on cooperative behavior in a one-shot PDG. (5) Spatial population expansion. Using an experimental PDG, Van Dyken et al. (83) concluded that spatial population expansion propels the evolution of cooperation via increasing the positive genetic assortment at population frontiers and via selecting for phenotypes that benefit the productivity of local demes. To our knowledge, however, no neuroimaging studies have examined the neural bases of the modulation of cooperativeness by these factors. To summarize, researchers have explored factors that affect game behaviors in the PDG from a number of perspectives. Being aware of the multiple sources of potential confounds is helpful for forming strict experimental designs and therefore facilitates a further understanding of the cognitive mechanisms involved in game behaviors and their neural substrates.

## Game Theory Paradigm: Research Applications and Implications in MDD

### Applications of the game theory paradigm in MDD

Improving social and interpersonal functioning is a key component of successful interventions for depression (84, 85). As we mentioned in the Section “Introduction”, one possible avenue for understanding social functioning in people with MDD is to let them accomplish tasks that involve cooperation, reciprocity, deception, and behavior adjustment relying on the decision behaviors of others. A game theory paradigm suits these requirements and many significant findings have been obtained for people with MDD.

Several studies administered the UG paradigm to MDD patients to explore deviations in their social-interaction behavior (86–91). The findings of these studies not only indicate impaired social decision-making in MDD, but also increase our understanding of the social cognition of depressives. Researchers have found that the altered performance in the UG by MDD patients can be explained from the perspective of social cognitive dysfunction, such as ToM deficits, negative cognitive schema, and reduced reward sensitivity. Previous studies have indicated that there are clear and consistent relationships between social cognition and aspects of social functioning (92, 93). Thus, investigating social cognition in depressives based on their performance in game theory paradigms can contribute to the understanding of social functioning in MDD.

In the game theory paradigm, the abilities associated with ToM play a critical role during interactions. The participants need to comprehend the intentions, beliefs, and wishes of others in social interactive tasks. Researchers have found that intact social cognition, especially ToM, plays an essential role in making a distinction between unfair proposals from computer proposers and human proposers in the UG (30). In one of our previous studies, we included human and computer offers in the UG and found aberrant decision-making behaviors in MDD patients when receiving unfair proposals from human and computer partners (91). In the human proposer condition, the participants needed to surmise the intentions of others, whereas in the computer proposer condition they did not. However, unlike healthy controls, the MDD patients were unable to respond discriminatively to unfair proposals from computer partners and human partners. Thus, it is possible that dysfunction in ToM made the depressed patients treat the human partner and the computer partner indiscriminately. However, the exact role of ToM deficits in the abnormal performance of MDD patients during the game theory paradigm can better be explored by using an interactive game theory paradigm or other paradigms, such as a mini-UG, which is an adapted version of UG to disentangle inequity aversion and intentionality considerations (94). We believe that the use of these game theory paradigms is a useful complement to the traditional methods for studying ToM.

Negative cognitive schema is a major characteristic of patients with MDD. To be specific, MDD patients are more sensitive to negative stimuli in their environment and tend to treat neutral or ambiguous stimuli as negative or as less positive. This negative cognitive bias has been observed in memory and attention, facial emotion processing, and the social and moral emotions of patients with MDD (95). In studies of game behavior, Gradin et al. (88) found that depressed patients reported decreased levels of happiness when facing “fair” offers in the UG in comparison to controls. Harlé et al. (89) found that depressed participants exhibited more negative emotional reactions to unfair offers. We also found that MDD patients perceived fairness differently from normal controls in our UG study, in that they had a tendency to judge an offer as less fair than the normal participants did (91). Thus, we conjecture that the negative cognitive schema of MDD also exists in social interactive contexts. Consistent with this speculation, we found that the acceptance rates of the depressed patients were lower than those of the controls (91). It is possible that the negative cognitive schema makes MDD patients pay more attention to negative cues such as inequitable offers than to reward cues, thus causing more rejections. Although the game behavior of people with MDD in the UG still needs to be investigated extensively, this negative schema cannot be ignored when predicting and explaining their behaviors.

Research has showed that depressed individuals display decreased reward sensitivity and that depression is associated with reduced activation in reward-related brain regions (96). In the UG, fair offers seem to activate reward-related brain regions, such as the striatum and the ventromedial PFC (14), and, accordingly, increasingly fair offers from a partner can be regarded as social rewards. Thus, the reward-related processing in people with MDD can be investigated by implementing UG tasks. Gradin et al. (88) investigated brain activation using fMRI in unmedicated, depressed participants. The results showed that, with an increase in offer fairness, the normal controls activated the nucleus accumbens and the dorsal caudate, regions that associate with processing social information and rewards (97). By contrast, the depressed participants did not activate these regions with increasing fairness. The participants with depression also exhibited a diminished response to increasingly unfair offers in the medial occipital lobe, a region that has been reported to be associated with early visual processing of social information (98). This study suggests that there are significant differences between depressed individuals and healthy controls in the neural substrates involved in processing social information, and reward. In addition, hyposensitive responses to reward appear to underlie a failure to maximize potential monetary earnings. In the UG, most studies also found that MDD patients showed statistically or numerically decreased acceptance rates compared with normal controls (86, 87, 90, 91), resulting in the subjects in the depressed group earning less money.

Despite these important findings about MDD, obtained by using the UG, it is noteworthy that the results were inconsistent, with studies discovering increased, unchanged, or decreased rejection rates to unfair proposals in depressed individuals (86–91). We speculate that the inconsistent findings in the UG behavior of patients with MDD may be due to the following confounding factors. The first is sample heterogeneity. In the study of Harlé et al. (89), the participants were undergraduate students and were drug-naïve patients and 4 out of 15 subjects were diagnosed subthreshold MDD (89). In the other studies, the patients were in a clinical population and most of them were taking antidepressant drugs. The inconsistent findings also suggest heterogeneity in the severity of symptoms. In accordance with this conjecture, we found a significantly negative relationship between the patients’ acceptance rates and the severity of their depression in our study (91). In addition, the mixed findings suggest a role for antidepressant drugs in the ultimatum bargaining behavior of depressed patients. It has been found that a kind of antidepressant can increase acceptance rates in healthy participants (99). In keeping with this, it seems likely that antidepressants should increase acceptance rates, but antidepressants may have a different influence on the brains of MDD patients compared with healthy participants. The effects of long-term antidepressants on the UG behavior of MDD patients should be investigated in the future. Furthermore, the task paradigms and experimental parameters have differed among the UG studies that involved MDD patients. Agay et al. (86) adopted a two-round UG paradigm (a demonstration round and a real round), but Destoop et al. (87) made the participants play the responder role first and then the proposer role with the same partner in each round. This procedure differed from that used in the other studies in which the participants only played as responders in all the rounds. Additionally, some experimental parameters, such as the number of trials, the stakes, and the response time limit, also varied between the studies. Thus, the experimental design may be a potential source of confounding factors that contributed to the inconsistent results.

To date, only a few studies have investigated the behaviors of MDD patients in the TG (100–102). In the study by Zhang et al. (102), all the participants played as trustees. In each trial, the participants received a request from the investor for a certain amount of the expected repayment and the participants had to decide whether to give more (altruistic act), the same, or less (deceptive act) than the expected amount. The money obtained in the trial was confiscated if the deceptive act was caught. The results showed that the depressed people made both fewer altruistic and fewer deceptive responses than the healthy participants in all the conditions. Furthermore, the specific behavioral pattern of the MDD patients was modulated by the task factors, including the risk of deception detection and the other players’ intentions (benevolence vs. malevolence). These results reflect the tendency of people with depression to be self-focused (102). It may be difficult for depressed patients to integrate information of both risk and other people’s intentions into social decisions. Using the same experimental design, Shao et al. (101) investigated the neural basis of MDD patients’ reduced tendency to make low-risk cheating choices. They found that when comparing brain activations during low-risk cheating with those during benevolent choices, the MDD patients exhibited weaker activations than the controls in the executive networks of the dorsolateral PFC and in the anterior insula and dorsal putamen, which have been implicated in value- and risk-based instrumental behaviors. They also obtained limited evidence that MDD patients showed abnormal IFG activity when making high-risk cheating choices. These findings have provided new theoretical insights into the neural mechanisms of risky decision-making processes in people with MDD in social contexts and may indicate possible functional deficiencies in the lateral PFC-striatal/limbic networks that are critical for risk-adaptive social responses. In addition, Cáceda et al. (100) further examined the role of gender in the potential impacts of depression on TG behavior. They found that depressed men showed more reciprocal behavior than healthy men, whereas this effect was absent between depressed and healthy women. In addition, suicidal ideation induced a gender-specific pattern of self-centered behavior (not giving after receiving): suicidal depressed men were less self-centered whereas suicidal depressed women were more self-centered. This study indicated that depression, particularly suicidal ideation, is related to a reversal of the gender-specific patterns of reciprocal behavior, suggesting the possibility of aberrancy in the regulation of the sex hormones. It is worth noting that the above findings need more studies to validate the findings, and, specifically, research is needed from the perspective of the investor.

The game behaviors of MDD patients in the PDG were also investigated. Hokanson et al. (103) used a modified PDG in which each player’s relative power was manipulated. The results indicated that the interactive pattern of depressives in the high-power role was relatively exploitive and non-cooperative, whereas depressives in the low-power role did not exhibit unique game behaviors. Haley and Strickland (104) found that depressed participants who experienced betrayal were more critical of their own decision on a subsequent interaction. Depressed, betrayed subjects also behaved more aggressively to their betraying partner than did non-depressed, betrayed subjects. These two early studies helped researchers to understand the impaired social-interactional approaches and cognitive schemata of depression. More recently, Surbey (105) investigated the relationship between depressive symptoms and cooperation as well as whether cognitive styles significantly influenced cooperative behavior. The results showed that the participants with more severe depressive symptom exhibited a significantly reduced intention to cooperate in the PDG. The hierarchical regression showed that individuals with heightened attributional optimism, or a tendency to attribute good events to more stable causes than negative events, cooperated more. In addition, Clark et al. (106) found a significantly negative correlation between an individual’s depressive symptoms and his/her performance in the prisoner’s dilemma. Pulcu et al. (107) also found that symptomatic patients defected significantly more often in the PDG. Thus, depressive symptoms were associated with an inability to sustain reciprocal cooperation. The findings from these studies contributed to furthering our understanding of the specific patterns of social behavioral changes associated with depression.

### Potential implications of the game theory paradigm in MDD

Building from the above neuroimaging exploration into the game theory paradigms, researchers studying social decisions have focused on identifying neurocognitive processes that may have uniquely evolved to guide social behavior. However, which aspects of the choice situations or which of the specific decision-making stages may be involved in social decisions in MDD remains unclear. Analyzing the different decision-making stages in social interactions will open a new avenue for studies of MDD. Ruff and Fehr (108) proposed three classes of situations that involve different targets and reference frames for social decision processes. The first class includes all situations in which an agent assesses how specific other individuals and their behavior affect his or her own well-being. The second class concerns situations in which an agent’s brain evaluates choice options and outcomes vicariously for others. The third class comprises situations in which an agent guides his or her behavior to comply with normative social principles. The specific choice situations were assessed for each of the three paradigms. In addition, the decision-making stage, which includes the choice of an appropriate action, an evaluation of the choice outcome, and learning from the outcome, was also assessed for each of the three paradigms and is discussed in this review. Researchers have reported that the decision process of the investor in the TG is a first class situation. Thus, an example of the choice, outcome, and learning stages for this situation can be deciding whether to invest money with someone, finding out that the other person defected, and learning about the other person’s trustworthiness (108). The UG and PDG, in which normative social principles concern fairness and cooperation, are examples of third class situations. An example of the decision stage for this situation could be altruistic punishment of norm violations, enjoying fair distributions/mutual cooperation, and changing the participant’s opinion to increase social conformity (108). Investigating the social decision-making stages of game behaviors in MDD patients can be expected to be helpful for understanding the behavioral characteristics of MDD in each stage and, further, for figuring out specific stages in which abnormal decision-making behavior occurs in the patients. Furthermore, developing new research paradigms based on these distinct decision-making stages may facilitate more targeted research on depression.

The predominant current medical view is that depression is a mental disorder (1). Great strides have been made by researchers and practitioners in diagnosing, treating, and understanding the prognosis of MDD patients in the past 50 years. Though the medical model of MDD as pathological is the majority view, some researchers have attempted to explain it from the perspective of evolutionary adaptation, with one approach arguing that depression itself is an adaptation. One of the evolution-related models is the adaptationist social navigation hypothesis (SNH) for MDD (109–111), which proposes that depression plays two complementary roles in dealing with particularly important and troublesome social problems. These roles are carried out by (1) a social rumination function and (2) a social motivation function (111). Because most instances of MDD appear to be initiated by adversity, evolutionary theories of MDD generally propose that sadness and low mood evolved as beneficial responses to adversity. Thus, evolutionary theorists hold that the social rumination function enables people in depressive situations to focus their limited cognitive resources on planning ways out of complex social problems. The social motivation function can, in turn, induce close social partners to provide problem-solving help and make concessions for the depressed person. On the one hand, some findings about depressives from game theory paradigms may be explained by this adaptive view. For example, the finding that depressed subjects exhibit lower acceptance rates of unfair offers and thus gain less money is very much in line with the expectations of the SNH, in that it proposes that depression (related to sadness) functions to resist behavior by the social network that tends to maintain the responder in an unfavorable (low profit) social niche. On the other hand, it is possible that game theory paradigms may be used to test predictions of the evolutionary adaptationist MDD hypotheses. Although evolutionary theory is a reasonably parsimonious account of the known facts about MDD, few of its predictions have been explored. In other words, adaptationist hypotheses have not yet been thoroughly tested. If the game theory paradigm can be thoughtfully implemented in ways that test the adaptationist hypotheses of MDD, it may reveal that MDD has an adaptive functional design. For example, a study could obtain initial measures of game behavior on subjects, then treat them using various interventions, including those recommended by the adaptationist SNH, and finally remeasure their responses to the same game. If MDD is an adaptation, individually meaningful practical progress toward solving the target problem should quickly “normalize” game behavior, or at least move it in that direction. Though the adaptationist theory of MDD is far from being a mature theory, exploring this evolutionary body of theory could make it more complete, opening up a whole new view to many in the field of MDD studies.

## Limitations of the Game Theory Paradigm

Game theory paradigms offer some real advantages over standard decision-making paradigms, not in the least that they embed actual, consequential, social interactions that allow investigation into complex processes, such as reputation, trust, equality, and cooperation. However, these paradigms have limitations and challenges that need to be addressed. First, as we have reviewed in the Section “Factors Modulating Game Behaviors and Their Neural Bases”, many contextual factors and experimental parameters can affect game behavior; thus, comparisons between studies are complicated. Second, game theory paradigms are relatively more complex than traditional non-social cognitive tasks. Therefore, the participants are vulnerable to the operation of the person who was conducting the experiment. But this effect can be lowered through standardizing the experimental procedures. In addition, interactions between individuals have often been strictly controlled in most previous studies. In other words, the decision-making process of the participants has been carried out in a closed laboratory. Most researchers believe that using these settings to conduct classic game theory paradigms is helpful for avoiding interference from irrelevant information with the individuals’ decision-making, but this has clearly weakened the ecological validity of these experiments. Future research should try to incorporate virtual reality techniques and hyperscanning to resolve the conflicts between experimental control and ecological validity. Virtual reality technology is a new research tool that can replicate, simulate, and represent the real world through its capacity for allowing the creation and control of dynamic three-dimensional, ecologically valid virtual environments (112). Based on the characteristics of the game theory paradigm, researchers can create an advanced form of human–computer interface that allows the user to “interact” with and become “immersed” in a computer-generated environment in a seemingly natural fashion. Hyperscanning is a technique that allows the simultaneous recording of the brain activity of different subjects during a social interaction (113). Using fMRI, electroencephalographic (EEG), and near-infrared spectroscopy (NIRS) devices, hyperscanning can study inter-brain correlations between the cerebral activities of a group of interacting subjects as a unique system. Such human–human interaction experimental designs seem quite similar to real-life social exchange and thus may have high ecological validity. The use of these techniques may give us a more comprehensive understanding of the motivation and psychological mechanisms involved in social decision-making. Furthermore, currently although a few researchers have utilized game theory paradigms to study the interaction between close members of a group (114), most of the experiments have concentrated on stranger interactions. In real-life situations, people are not really designed to interact much with total strangers in one-off situations. For people in real-life situations, maintaining and building reputation matters. Researchers should give more considerations to the use of close friends or family members to increase the ecological validity of game theory paradigms in the future.

## Perspectives

In the present paper, we summarized the current findings for the healthy population and for depressed patients obtained by using a game theory paradigm, especially the UG. We believe that game theory, through its interdisciplinary approach and its particular combination of methods that allows the precise mapping of social behaviors across multiple levels of exploration, can provide a new tool to bridge the gap between neurobiological research and clinical studies. Even more important is that these studies suggest the possibility that the altered game behaviors and brain activities in neuropsychiatric disorders could be useful biomarkers, providing information on the diagnosis, therapy evaluation, and prognosis of MDD and its subtypes, if any. But various challenges have to be solved to realize this potential. Some future prospects that need to be explored are proposed at the end of this paper.

### Biological bases of social dysfunction in MDD

One of the current challenges is our poor understanding of the biological basis of the altered decision behaviors in MDD. In this section, we express our opinion about ways to address this challenge in order to elucidate the underlying biological mechanisms of social dysfunction in MDD.

A potential direction would be to continue to explore the neural mechanisms underlying the altered social behaviors in MDD. Neuroimaging studies of game behaviors in MDD are still rare, and the preliminary findings need to be validated by a number of future studies. Neuroimaging techniques can help identify neural differences between depressed participants and controls and can further locate brain regions that may be linked to impairments in the social interactions of depressives. With the help of non-invasive brain stimulation techniques, such as transcranial magnetic stimulation (TMS) and tDCS, the function of target brain regions may be understood by disrupting activity in the specific brain region and observing the behavioral changes. Previous studies applying low-frequency TMS and tDCS have demonstrated that the right, but not the left, dorsolateral PFC plays a key role in implementing fairness-related behaviors in the UG (115, 116). This result illustrates the importance of TMS and tDCS in understanding the neural basis of human decision-making. By combining repetitive TMS with fMRI, Baumgartner et al. (115) also found that a prefrontal network, the activation of the right dorsolateral PFC and posterior ventromedial PFC, and the connectivity between them, facilitates normal subjects’ willingness to reject unfair offers. Thus, future studies should pay attention to the functional interactions between the brain regions involved in games from the perspective of brain networks. This type of research is important for understanding the neurobiological mechanisms of complex social behaviors in both healthy individuals and those with MDD. Additionally, structural abnormalities in the brains of patients with MDD have been observed in the cortical and subcortical regions (for a review, see Ref. (117)). Future studies need to clarify and verify whether structural abnormalities in certain brain regions are related to the altered game behavior of people with MDD.

Another direction would be to explore the physiological mechanisms for the altered game behavior observed in MDD from the perspective of neurotransmitters. Serotonin deficiency has been found in MDD patients (118, 119). Recent studies confirmed that serotonin plays an important role in game behaviors (99, 120–122). Healthy participants rejected more unfair offers in the UG after their serotonin levels were lowered using acute tryptophan depletion and accepted more unfair offers after their serotonin levels were enhanced with citalopram (99, 120). Another study found that individuals with a low level of serotonin transport in the dorsal raphe nucleus were more likely to be intolerant of unfair offers, and thus more likely to engage in rejecting unfair offers (122). In addition, neurotransmitters, such as serotonin and OT, are also closely correlated with modulations in game behavior in the TG and PDG in healthy populations (71, 81, 82). However, it is not clear whether the changes in neurotransmitters observed in MDD play a critical role in the patients’ abnormal game behavior. Thus, investigating the function of neurotransmitters in game behaviors can be beneficial for illuminating the physiological mechanisms underlying the altered game behaviors observed in MDD.

Furthermore, exploring the genetic basis is another avenue for advancing our understanding of the social dysfunction identified by using a game theory paradigm with MDD patients. Cesarini et al. (123) summarized the findings from a research study of the heritability of behavior in some widely used economic games, including the UG and TG. The results suggested that 42% of the variation in the subjects’ rejection behaviors in the UG can be explained by genetic effects (124). In addition, the heritability of trust is estimated to be 20% in Sweden and 10% in the U.S and the heritability of trustworthiness is estimated to be 18% in Sweden and 17% in the U.S (125). These results suggest that humans are endowed with genetic variations that influence the decisions in game theory tasks in healthy populations. MDD has been demonstrated to be heritable (126, 127). However, due to a lack of twin and family studies that focus on social functioning, it is unclear whether the altered game behavior in MDD patients is also influenced by abnormalities in their genetic composition. Increased use of genetic techniques is needed to clearly elucidate how genes, the brain, and the disorder interplay.

### Trait-state distinction for social dysfunction in MDD

Despite the current knowledge about the applications of the game theory paradigm in MDD, it remains unclear whether this social dysfunction represents a mutable, temporary state or a relatively stable trait marker. All of the current studies examining game behavior are cross-sectional studies, which only compared the decision behaviors in MDD patients with those in healthy controls. Additional studies that investigate the game behavior in MDD from a variety of perspectives are urgently needed to address this issue more clearly. For example, longitudinal studies, which can help track the trajectory of disorders; studies of treatment effects, which can promote our understandings of implications of the pharmacology and pathophysiology of MDD; studies using drug-naïve patients, which can distinguish the effects of medication on social behaviors from the effects of the disease *per se*; and studies recruiting relatives who are at a higher risk of developing a depressive disorder, which can provide valuable insight on “trait” characteristics of the disorder, will be essential for further advancing our understanding of these diseases and identifying trait-state distinctions for future clinical use. Only by a systematic exploration using extensive research can we conclude that an altered game behavior might serve as a prodromal predictor of susceptibility to MDD.

### Specificity of game behavior in MDD

Applications of a game theory paradigm in other mental disorders have also found impaired game behaviors in populations, such as schizophrenia, anxiety, and bipolar disorder. In order to apply a game theory paradigm to clinical use with MDD patients, the specific characteristics of the game behavior must be distinguishable between MDD and other disorders. A number of studies of game behaviors in MDD have been summarized in this paper. A brief outline of the major findings from a game theory paradigm in other mental disorders follows. (1) Schizophrenia. Recently, several researchers have done studies using schizophrenic patients playing the UG (86, 128–130). As proposers, these patients made more hyper-fair offers, the same rate of fair offers, and many fewer unfair offers compared with the controls (86). Similarly, using a sample of students, the participants who had higher schizotypal scores tended to offer more money (129). As responders, schizophrenic patients accepted a greater proportion of unfair offers and had lower rates of rejection of fair offers (128). Yet, the rejection rate increased as the offers became less fair, just as in healthy populations (130). The sample of students with high schizotypal scores presented the same behavioral pattern (129). In summary, these results show that schizophrenic patients had aberrant behavior by proposing more fair offers and accepting more unfair offers. (2) Anxiety disorder. Patients with MDD often display symptoms of anxiety. Previous studies also found that patients with an anxiety disorder showed altered game behavior in game theory tasks. Anxious patients accepted significantly more unfair offers than normal controls in the UG (131). Another study found that participants with a high level of trait anxiety rejected more computer-proposed inequitable offers than did those with a low level of trait anxiety. Moreover, the skin conductance response to inequitable offers was correlated with the level of anxiety in the high level of trait anxiety group, but not in the low level of trait anxiety group (132). A follow-up ERP study revealed that the high-anxiety group showed a larger feedback-related negativity when facing unequal offers than equal ones, and a larger P300 when facing offers from a human than from a computer, but these effects were absent in the low-anxiety group (133). In brief, people differ in their levels of anxiety, and patients with anxiety disorders showed distinctive behavior patterns during social decision-making. (3) Bipolar disorder. It is a severe condition typically characterized by manic and depressive episodes. Duek et al. (134) examined the UG behavior in patients with bipolar disorders who were currently euthymic (specifically, they had not been either depressed or manic for at least 1 month) and found that these patients rejected more of the moderately unfair offers than did healthy controls, a finding which was similar to those in MDD patients (90, 91). Future studies are needed to determine the similarity and differences between the behavioral characteristics of MDD patients from those in patients with bipolar disorders in game theory tasks. We believe that the best way to determine the specificity of game behavior in MDD patients may be by using neuroimaging paired with game-theoretic probes.

In summary, MDD patients have problems and difficulties with their interactions with others and their integration into society. Despite an increasing interest in research into social cognition in MDD, most research carried out in this realm has used non-interactive tasks, which do not capture the dynamic and unique nature of the social interactive processes. Utilizing an interactive game theory paradigm will allow us to develop clinical applications that are oriented to measuring and improving social functioning and thus provide a powerful tool for investigating social-interaction impairments in MDD. The research examining altered game behaviors and brain activities in MDD may be able to provide cues for identifying potential biomarkers for the diagnosis, therapy evaluation, and prognosis of MDD. To achieve this, more effort is needed to clearly elucidate the neural, physiological, and genetic bases of social dysfunction in MDD. Furthermore, the trait-state distinction for social dysfunction in MDD needs to be determined, and the specificity of game behavior in MDD also needs to be clarified.

## Conflict of Interest Statement

The authors declare that the research was conducted in the absence of any commercial or financial relationships that could be construed as a potential conflict of interest.
